# Oxidative Stress Signaling and Regenerative Responses in a Larval Zebrafish Model of Retinal Light Damage

**DOI:** 10.3390/antiox15030348

**Published:** 2026-03-10

**Authors:** Ignacio Babiloni-Chust, Luigi Donato, Samuele Sartori, Matthias Carl, Darin Zerti, Carmela Rinaldi, Vincenzo Flati, Marco Feligioni, Rosalia D’Angelo, Rita Maccarone, Lucia Poggi

**Affiliations:** 1 Department of Cellular, Computational and Integrative Biology (CIBIO), University of Trento, 38123 Trento, Italy; i.babilonichust@unitn.it (I.B.-C.); samuele.sartori@unitn.it (S.S.); matthias.carl@unitn.it (M.C.); 2 Department of Biomedical and Dental Sciences and Morphofunctional Imaging, University of Messina, 98125 Messina, Italy; luigi.donato@unime.it (L.D.); carmela.rinaldi@unime.it (C.R.); rosalia.dangelo@unime.it (R.D.); 3 Department of Biomolecular Strategies, Genetics and Cutting-Edge Therapies, I.E.ME.S.T.—Istituto Euro Mediterraneo di Scienza e Tecnologia, 90139 Palermo, Italy; 4 Department of Biotechnological and Applied Clinical Sciences, University of L’Aquila, 67100 L’Aquila, Italy; darin.zerti@univaq.it (D.Z.); vincenzo.flati@univaq.it (V.F.); rita.maccarone@univaq.it (R.M.); 5 Fondazione European Brain Research Institute (EBRI) Rita Levi-Montalcini, 00161 Rome, Italy; m.feligioni@ebri.it; 6 Department of Neurorehabilitation Sciences, Casa di Cura IGEA, 20144 Milan, Italy

**Keywords:** zebrafish (*Danio rerio*), light-induced retinal damage (LIRD), phototoxicity, LIRD, retinal regeneration, Müller glia, oxidative stress, *N*-acetylcysteine (NAC), antioxidants, transcriptomics

## Abstract

The zebrafish (*Danio rerio*) is a widely used model for studying retinal regeneration. In adults, light-induced retinal damage (LIRD) serves as an environmental phototoxic stressor that induces photoreceptor degeneration and regenerative responses, whereas larval models remain comparatively underexplored. In this study, we validate a larval LIRD paradigm as a versatile system for studying acute phototoxic injury and early regeneration-associated transcriptomic responses. Using high-throughput RNA sequencing, we profiled retinal transcriptional changes 48 h post-LIRD and complemented these findings with targeted pharmacological modulation of redox signaling. Larval LIRD induced robust activation of canonical apoptotic and regeneration-associated pathways, recapitulating key features of adult LIRD models while engaging previously underexplored gene-regulatory networks. Among these, pathways related to oxidative stress responses, antioxidant enzymes, and oxygen metabolism were prominently regulated. Functional attenuation of oxidative stress using the *N*-acetylcysteine reduced phototoxic injury-induced apoptosis and proliferation, while inflammatory markers remained largely unaffected. Conversely, subtoxic intra-retinal hydrogen peroxide exposure was sufficient to induce proliferative markers without eliciting apoptosis response. At the signaling level, modulation of oxidative stress influenced components of growth-associated signaling pathways activated during early injury response. Together, these findings support a role for oxidative stress as a key component of early injury-associated signaling in larval retinal regeneration. This study integrates histological, transcriptomic, and pharmacological analyses to interrogate early regenerative programs and provides a comprehensive transcriptomic resource for exploring redox-associated mechanisms in retinal injury and repair.

## 1. Introduction

The zebrafish (*Danio rerio*) retina exhibits a regenerative capacity absent in mammals, making it a powerful system for investigating the mechanisms underlying successful neuronal repair [1,2]. Following injury, Müller glia (MG), the principal glial cells of the retina, re-enter the cell cycle, dedifferentiate into a progenitor-like state, and generate multipotent progenitors capable of differentiating into all major retinal neuron types [3,4,5]. Among the commonly used injury paradigms is light-induced retinal damage (LIRD), in which photoreceptors rapidly degenerate due to excess of photo-oxidative stress [6,7,8,9,10,11,12,13,14,15,16,17]. Building on this and other retinal injury paradigms, numerous studies have identified molecular programs that drive MG reprogramming and neuronal regeneration, highlighting coordinated interactions among inflammatory signaling, chromatin remodeling, and transcriptional regulation [18,19,20,21,22,23,24,25,26,27,28,29,30,31,32]. While inflammatory pathways have been extensively characterized and functionally investigated in this context [18,20,28], comparatively less attention has been directed toward defining the contribution of oxidative stress-associated signaling beyond its established role in mediating photoreceptor damage. Cross-species single-cell and bulk transcriptomic comparisons have further delineated conserved and divergent aspects of MG activation across zebrafish, chick, and mouse retinae, particularly in adult injury models [33,34]. However, the earliest molecular events occurring within 48–72 h after injury remain undercharacterized, despite their crucial role in establishing a pro-regenerative transcriptional landscape.

Larval zebrafish provide unique advantages for dissecting such early responses. Their optical transparency, rapid development, small size, and compatibility with high-throughput imaging, sequencing and drug screening enable systematic interrogation of acute molecular dynamics in vivo. While LIRD has been extensively used in adult zebrafish to study photoreceptor injury and MG activation [8,13,24,26,29,35,36], fewer studies have examined larval stages [25,37], and a comprehensive characterization of transcriptional changes in larvae is lacking. This gap limits the adoption of larval LIRD as a scalable discovery platform for regenerative biology.

In this study, we performed LIRD on pigmented zebrafish larvae at 4 days post-fertilization (4 dpf) and profiled retinal responses 48 h post-injury using a multimodal experimental approach. By combining histological analyses, immunohistochemistry, transcriptomics, and targeted pharmacological interventions, we define acute cellular and molecular landscapes following phototoxic damage. Alongside canonical players, we identified coordinated regulation of several underexplored injury-associated pathways. Notably, oxidative stress and reactive oxygen species (ROS)-related gene networks were prominently regulated, reinforcing their role as essential mediators in the acute injury response.

The role of ROS has been highlighted in different developmental and regenerative contexts [38,39,40,41,42,43]. However, their functional contribution to zebrafish retinal regeneration particularly remains insufficiently defined. To explore the contribution of oxidative stress to the larval LIRD induced alterations, we combined controlled hydrogen peroxide (H_2_O_2_) exposure with pharmacological redox modulation using the known ROS scavenger *N*-acetylcysteine (NAC). NAC attenuated apoptotic signaling and suppressed MG proliferation, without markedly altering inflammatory gene expression, whereas subtoxic H_2_O_2_ enhanced proliferation in the absence of apoptosis. Together, these findings indicate that ROS functions as a modulatory cue in early MG activation and regenerative priming and underscore the pharmacological tractability of the larval LIRD system for in vivo pathway interrogation.

## 2. Materials and Methods

### 2.1. Zebrafish Husbandry, LIRD Paradigm, and NAC Treatment

All zebrafish procedures were conducted in accordance with the institutional guidelines of the University of Trento and approved by the Italian Ministry of Health (authorization no. 707/2025-PR). Wild-type TU and *Tg(fli:gal4;UAS:mCherry)* zebrafish were maintained under standard laboratory conditions at 28 °C on a 14:10 h light/dark cycle. Embryos were collected in Petri dishes and incubated in the dark at 28 °C in 1× E3 medium.

For the light-induced retinal damage (LIRD) paradigm, groups of 50 embryos at 4 dpf were transferred to a beaker containing 1× E3 medium without methylene blue. The beaker was covered with aluminum foil to enhance light reflection and exposed to intense bright light (50,000 lux) for 3.5 h. Illumination was delivered simultaneously from above and below using a stereomicroscope system (SteREO Discovery.V8, Carl Zeiss, Oberkochen, Germany) equipped with a dual-arm fiber-optic halogen light source (Leica KL 1500 LCD, Leica, Wetzlar, Germany; Osram 64634 HLX lamp, Osram, Munich, Germany). Temperature was continuously monitored and maintained at 28 °C throughout the exposure. Following LIRD, embryos were returned to dark conditions for recovery. At 48 h post-LIRD, embryos were anesthetized with tricaine (MS222, 160 mg/L) and retinae were dissected under a stereomicroscope for downstream analyses.

A single concentration of NAC (100 μM) was selected as non-teratogenic dose effective for modulating antioxidant defenses, based on established zebrafish protocols and preliminary toxicity assays [44,45]. At this concentration, no significant alterations in larval morphology, heart rate, or locomotor activity were observed. NAC solutions were freshly prepared in 1× E3 medium for each experiment, and pH-adjusted to 7.4 to prevent acidic stress. Immediately after LIRD, groups of 50 embryos were transferred to Petri dishes containing E3 supplemented with 100 µM NAC and allowed to recover under dark conditions. After 48 h, retinae were collected and processed for WB or RT-qPCR analyses.

### 2.2. Immunohistochemistry (IHC), Image Acquisition

Larvae were fixed in 4% paraformaldehyde (PFA) in PBS overnight at 4 °C with gentle agitation. For cryoprotection, samples were immersed in 30% sucrose overnight at 4 °C, embedded in FSC 22 Frozen Section Media, and sectioned at 15 μm using a Leica CM 1850 UV cryostat, (Leica, Wetzlar, Germany). Sections were collected on Superfrost Ultra Plus slides. To minimize non-specific binding, sections were incubated in blocking buffer (10% goat serum, 1% BSA, and 0.8% Triton X-100 in PBS) for at least one hour at room temperature. Primary antibodies (Supplementary Table S1) were diluted in blocking buffer and incubated overnight at 4 °C. Following three 10 min washes in PBST (PBS + 0.1% Triton X-100), secondary antibodies were applied in blocking buffer for one hour at room temperature. For PCNA immunostaining, heat-induced antigen retrieval was performed by boiling slides in 10 mM sodium citrate (pH 6.0) with 0.01% Tween-20 for 20 min prior to the blocking step. Slides were mounted using VECTASHIELD Antifade Mounting Medium with DAPI (Vector Laboratories, H-1200, Newark, CA, USA) and cured for 48 h before imaging. Confocal microscopy was performed on a Leica SP8 system (LAS AF Software 2.6.0) using an HC PL APO 63×/1.30 Oil CS2 objective. Images were acquired at a resolution of 2048 × 2048 pixels (speed: 200 Hz, step size: 1 μm), with z-stacks spanning the entire retinal thickness. For Caspase-3 (Casp3) imaging, a Zeiss Axio Imager M2 microscope (Carl Zeiss, Oberkochen, Germany) with a 20× objective was utilized. Image processing and quantitative measurements were conducted using Fiji (ImageJ v 1.54q). To assess the structural integrity of the photoreceptor compartment, morphometric analysis was performed on Zpr1/DAPI-stained cryosections.

### 2.3. Morphometric Analysis and Quantifications

The Photoreceptor Cell Layer (PCL) thickness was defined as the longitudinal distance from the basal-most side of the DAPI-stained nuclei, where the synaptic pedicle was evident, to the furthest apical extent of the Zpr1-positive signal. To ensure data robustness, measurements were averaged across three distinct confocal planes (z-sections) per specimen, with three independent measurements taken at equal distances per plane. The resulting arithmetic mean served as the representative value for each biological replicate (*n* = 8).

### 2.4. H_2_O_2_ Intra-Retinal Injection

For ROS induction, larvae at 4 dpf were anesthetized with MS222 and positioned dorsally, with one side laying on the edge of a glass slide. Using a glass capillary (Harvard Apparatus; 30-0019) connected to a microinjector (FemtoJet 4X Microinjector, Eppendorf, Hamburg, Germany), 3 nL of a 100 µM H_2_O_2_ solution was injected into each retina. Phosphate-buffered saline (PBS) was used as a vehicle control. After injection, larvae were allowed to recover under dark conditions at 28 °C in a Petri dish and E3 embryo medium. At 48 h post-injection, retinae were collected and processed for Western blot or RT-qPCR analyses.

### 2.5. Western Blot

Pools of 50 retinae were collected, washed in cold PBS, and lysed in Laemmli lysis buffer. Protein extracts were resolved on 8–16% Mini-PROTEAN TGX precast gels (Bio-Rad) and transferred onto nitrocellulose membranes (Bio-Rad, 1620264, Feldkirchen, Germany). Membranes were blocked for 1 h at room temperature in either 5% non-fat milk or 5% BSA with gentle shaking, followed by overnight incubation with primary antibodies at 4 °C.

After three washes in Tris-buffered saline with Tween-20 (TBST), membranes were incubated with HRP-conjugated secondary antibodies for 1 h at room temperature. Signal detection was performed using SuperSignal West Femto chemiluminescent substrate (Thermo Scientific, 34075, Eugene, OR, USA) and was acquired on a Bio-Rad ChemiDoc XRS imaging system. Densitometric analyses were performed using Image Lab software (version 4.1, Bio-Rad Laboratories).

A complete list of primary and secondary antibodies used for Western blotting, including working concentrations, is provided in Supplementary Table S1.

### 2.6. RNA Isolation, Quality Control, and RT-qPCR

Total RNA was extracted using TRIzol reagent (Invitrogen, 15596026, Waltham, MA, USA) according to the manufacturer’s instructions. RNA pellets were resuspended in RNase-free water and quantified using the Qubit RNA HS Assay Kit (Thermo Fisher Scientific, Q32852, Eugene, OR, USA). RNA integrity was assessed using an Agilent 2100 Bioanalyzer (Agilent Technologies, Santa Clara, CA, USA); with RNA 6000 Nano Chips. Only samples with an RNA Integrity Number (RIN) ≥ 7 and OD260/280 ratios between 1.9 and 2.1 were used for downstream analyses.

Reverse transcription was performed using the RevertAid First Strand cDNA Synthesis Kit (Thermo Fisher Scientific, K1622) with random hexamer primers. Quantitative real-time PCR (RT-qPCR) was carried out on a CFX96 Real-Time PCR Detection System (Bio-Rad Laboratories, 3600037) using iTaq Universal SYBR Green Supermix (Bio-Rad, 1725121) in 10 µL reaction volumes, following the manufacturer’s instructions.

Data were analyzed using CFX Manager software (version 1.6, Bio-Rad) and expressed as relative gene expression levels using the 2^−ΔΔCt^ method, normalized to control samples. The housekeeping gene *ube2a* was used for normalization. All reactions were performed in technical triplicates. Primer sequences are listed in Supplementary Table S2.

### 2.7. Library Preparation and RNA Sequencing

Poly(A)+ mRNA was isolated from total RNA using oligo-dT magnetic beads. Strand-specific sequencing libraries were prepared using the Watchmaker RNA Library Prep Kit (Twist Bioscience; 7BK0001-096, Boulder, CO, USA) according to the manufacturer’s instructions, including first- and second-strand cDNA synthesis, end repair, adapter ligation, and incorporation of unique dual indices for sample multiplexing.

Final libraries were assessed for concentration, quality, and insert size distribution (target range: 300–400 bp) using a Fragment Analyzer system. Equimolar pools of indexed libraries were sequenced on an Illumina NovaSeq 6000 platform (S4 flow cell, paired-end 2 × 150 bp), yielding an average depth of approximately 80 million reads per sample.

### 2.8. Primary Bioinformatic Analysis: Read Processing and Alignment

Initial quality control of raw FASTQ files was performed using FastQC v0.12.1. Adapter sequences and low-quality bases were removed using Trimmomatic v0.39 (ILLUMINACLIP:2:30:10, SLIDINGWINDOW:4:20, MINLEN:36). High-quality reads were aligned to the *Danio rerio* reference genome (GRCz11, Ensembl release 108) using STAR aligner v2.7.10a with default parameters and two-pass mapping enabled to improve splice junction detection.

### 2.9. Gene Quantification and Differential Expression Analysis

Aligned reads were quantified using two complementary approaches: (i) featureCounts (Subread v2.0.3) with exon-level summarization and strand-specific counting; (ii) CLC Genomics Workbench (Qiagen, v25.0.0), which was used to assess overall mapping efficiency, transcript-level abundance expressed as transcripts per million (TPM), fusion events, and transcript diversity through its RNA-Seq Analysis Module.

Raw count matrices generated by featureCounts were imported into R (v4.3.1) for differential expression analysis using DESeq2 (v1.48.0). Genes with an absolute log_2_ fold change > 0.585 and a Benjamini–Hochberg-adjusted *p*-value (false discovery rate, FDR) < 0.05 were considered significantly differentially expressed (DEGs).

Model performance and data quality were assessed by inspecting dispersion estimates, MA plots, and principal component analysis (PCA). PCA was used as an exploratory quality control step to evaluate sample-to-sample variability based on global gene expression profiles (log_2_ TPM). Samples behaving as multivariate outliers and failing to cluster with their biological replicates were excluded from downstream analyses. Only samples passing quality control were retained for PCA visualization and differential expression analysis.

### 2.10. Functional Annotation and Pathway Enrichment Analysis

Functional enrichment analyses of DEGs were performed using multiple independent tools to ensure robustness and cross-validation of enriched terms, including g:Profiler (g:GOSt; default multiple-testing correction), Enrichr (KEGG, Reactome, WikiPathways), DAVID Bioinformatics Resources 2023 (v6.8), and ReactomePA v1.54.0 (R package for curated Reactome pathway enrichment).

Gene Ontology (GO) categories, including Biological Process (BP), Molecular Function (MF), and Cellular Component (CC), were analyzed. Pathways with a corrected *p*-value < 0.05 identified by at least two independent tools were retained for interpretation. Canonical regeneration-associated pathways (e.g., Jak/Stat, Notch, Wnt) were evaluated in the context of established zebrafish retinal regeneration literature, whereas less-characterized pathways were cross-referenced with PubMed and ZFIN to assess prior biological annotation.

### 2.11. Transcript-Level Analysis and Isoform Dynamics

Transcript abundance and alternative splicing were assessed using StringTie2 (v2.2.1) with reference-guided transcript assembly. In parallel, CLC Genomics Workbench was used to visualize exon usage, transcript structure, and potential fusion events across conditions.

Differential transcript usage (DTU) was evaluated using the DEXSeq framework, enabling the identification of isoform-level regulation in regeneration-associated genes, including *sox2*, *ascl1a*, and *stat3*.

### 2.12. Data Visualization and Statistical Analysis

Data visualization was performed in R using the following packages: ggplot2 (v3.4.2), ComplexHeatmap (v2.14.0), EnhancedVolcano (v1.18.0), clusterProfiler (v4.8.1), and GOplot (v1.0.2). Heatmaps and volcano plots were standardized across contrasts. Pathway interaction networks were generated using Cytoscape (v3.10.0) with the ClueGO plugin. All raw and processed RNA-seq data have been deposited in the NCBI Gene Expression Omnibus (GEO) under accession number GSE313277.

For non-transcriptomic analyses, statistical analyses were performed using GraphPad Prism 8.0 (GraphPad Software, La Jolla, CA, USA). Data normality was assessed using the Shapiro–Wilk test. Parametric data were analyzed using Student’s *t*-test, whereas non-parametric data were analyzed using the Mann–Whitney test, as specified in the corresponding figure legends. Data are presented as mean ± s.d., and statistical significance was defined as *p* ≤ 0.05. Schematics were created in biorender.com.

## 3. Results

### 3.1. Light-Induced Retinal Damage Elicits Early Degeneration- and Regeneration-Associated Responses in Zebrafish Larvae

Light-induced retinal damage (LIRD) was induced in pigmented larvae at 4 dpf by exposure to high-intensity white light (50,000 lux for 3.5 h). Cellular and molecular signatures associated with early degenerative and regeneration-related responses were then assessed 48 hours-post-light-induced retinal damage (HPL) using immunohistochemistry, Western blot analysis (WB), and transcriptomic profiling (Supplementary Figure S1A).

**Figure 1:**
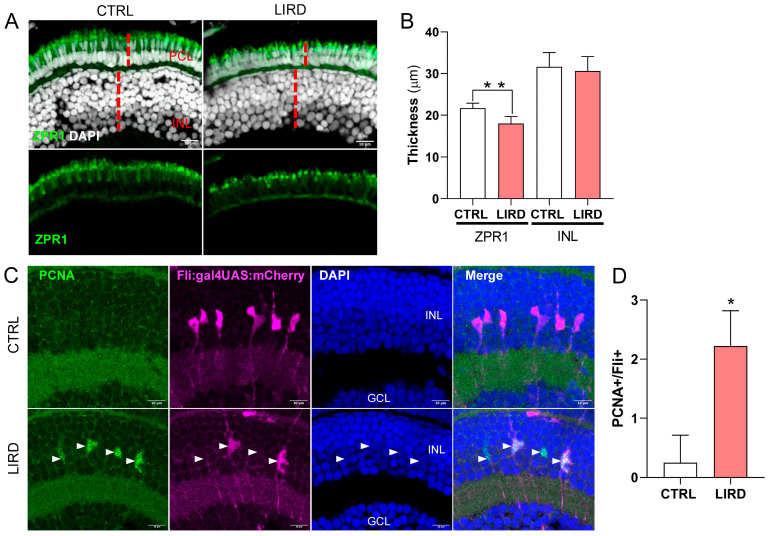
Larval LIRD triggers photoreceptor dysmorphology and MG-derived progenitor proliferation. (**A**) Representative confocal images (single z-stacks) of control (CTRL) and LIRD retinae immunolabeled with Zpr1 (red/green cone marker, green) and DAPI (nuclei, grey). Red dashed vertical lines depict the measured thickness of the inner nuclear layer (INL) and the photoreceptor cell layer (PCL). (**B**) Quantification of PCL and INL thickness in µm (*n* = 8 retinae per condition; each value represents the average of 9 measurements per retina). (**C**) Representative confocal images (single z-stacks) of CTRL and LIRD *Tg(fli:gal4;UAS:mCherry)* transgenic retinae (magenta), immunolabeled for PCNA (green) and counterstained with DAPI (blue). Arrowheads indicate fli:mCherry^+^/PCNA^+^ double-positive cells within the INL. (**D**) Quantification of fli:mCherry^+^/PCNA^+^ cells per retinal section (*n* = 8 retinae per condition). Data are presented as mean ± S.D. Statistical significance was assessed using an unpaired two-tailed Student’s *t*-test for (**B**) and a Mann–Whitney test for (D); * *p* ≤ 0.05, ** *p* ≤ 0.01. Scale bars = 10 µm. GCL, ganglion cell layer, INL, Inner nuclear layer, PCL Photoreceptor cell layer.

To assess photoreceptor integrity following LIRD, we performed immunofluorescence staining for Arrestin 3a (Arr3a/Zpr1), a marker of red/green double cone photoreceptors [46]. In control retinae, photoreceptor cell nuclei formed a regularly spaced and organized layer, with Zpr1 labeling defining a continuous domain extending from the synaptic pedicle to the apical inner segment (Figure 1A, CTRL). In contrast, LIRD-treated retinae exhibited significant photoreceptor dysmorphology, with the nuclei appearing disorganized and more densely packed compared to controls (Figure 1A, LIRD). This was accompanied by a marked reduction in the apico-basal thickness of the Zpr1-positive domain, suggesting structural collapse of the photoreceptor compartment (Figure 1A,B). These changes are consistent with acute early stages of the retinal stress response.

To evaluate early proliferation-associated responses in the central retina, we examined the expression of proliferating cell nuclear antigen (PCNA). To specifically identify proliferating MG, we used the transgenic reporter line Tg(fli1:gal4;UAS:mCherry) [47] Validation of this reporter line confirmed that 78.2 ± 8% of Glutamine Synthetase (GS)-positive MG in the central retina of 6 dpf larvae co-expressed mCherry (Supplementary Figure S2). Beyond molecular markers, these mCherry+ cells exhibited the hallmark bipolar morphology of MG, with apical processes extending toward the photoreceptor layer and basal end feet at the inner limiting membrane (Figures S2 and 1C) [6,35]. This high degree of overlap and morphological consistency supports the reliability of this reporter line for identifying the MG population in the larval zebrafish retina.

Under physiological conditions at 6 dpf, the central retina is largely devoid of PCNA expression, aside from a few dividing late precursors [48] Consistently, mCherry-positive MG in control larvae rarely expressed PCNA (Figure 1C,D CTRL). Following LIRD, we observed robust induction of PCNA within the mCherry-positive MG population (Figure 1C,D LIRD), consistent with injury-induced cell cycle re-entry prior to the loss of differentiated markers [6,35] These findings demonstrate that LIRD triggers reactive proliferation of MG, consistent with their established role as intrinsic retinal stem cells during regeneration.

**Figure 2:**
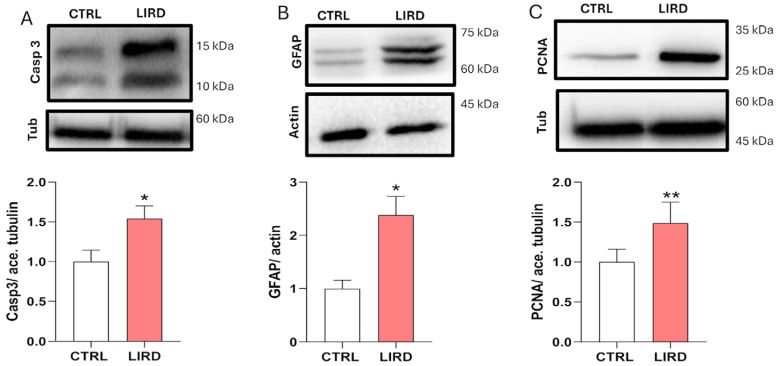
Larval LIRD induces apoptotic and regeneration-associated molecular responses. (**A**) Representative WB (top) showing cleaved caspase-3 (Casp3), with acetylated tubulin (ace. tub) used as a loading control. Densitometric quantification (bottom) shows Casp3 levels normalized to control averages (N = 5 biological replicates). (**B**) Representative WB (top) showing glial fibrillary acidic protein (GFAP), with actin used as a loading control. Densitometric quantification (bottom) normalized to control averages (N = 4 biological replicates). (**C**) Representative WB (top) showing proliferating cell nuclear antigen (PCNA), with acetylated tubulin used as a loading control. Densitometric quantification (bottom) normalized to control averages (N = 5 biological replicates). Data are presented as mean ± SEM. Statistical significance was assessed using an unpaired two-tailed Student’s *t*-test. * *p* ≤ 0.05, ** *p* ≤ 0.01.

WB analysis further confirmed molecular responses associated with LIRD-induced injury (Figure 2). Cleaved caspase-3 levels were significantly elevated (~1.5-fold) compared to controls (Figure 2A), consistent with increased caspase-3 immunolabeling (Supplementary Figure S3) and reflecting activation of caspase-dependent apoptotic pathways, in agreement with previous reports in phototoxic retinal injury models [25] At the same time, WB analysis revealed marked upregulation of GFAP (~2.4-fold) and PCNA (~1.5-fold) protein levels following LIRD (Figure 2B,C), in line with global MG activation and increased proliferative activity in the injured retina, in line with the MG-specific proliferation demonstrated by PCNA immunohistochemistry (Figure 1).

Collectively, these results demonstrate that LIRD induces coordinated apoptotic, gliotic, and proliferative responses in the zebrafish larval retina, recapitulating hallmark responses observed in established adult retinal injury models.

### 3.2. Transcriptomic Analysis Delineates Canonical Regeneration Programs Alongside Underexplored Metabolic and Redox Stress Responses

**Figure 3:**
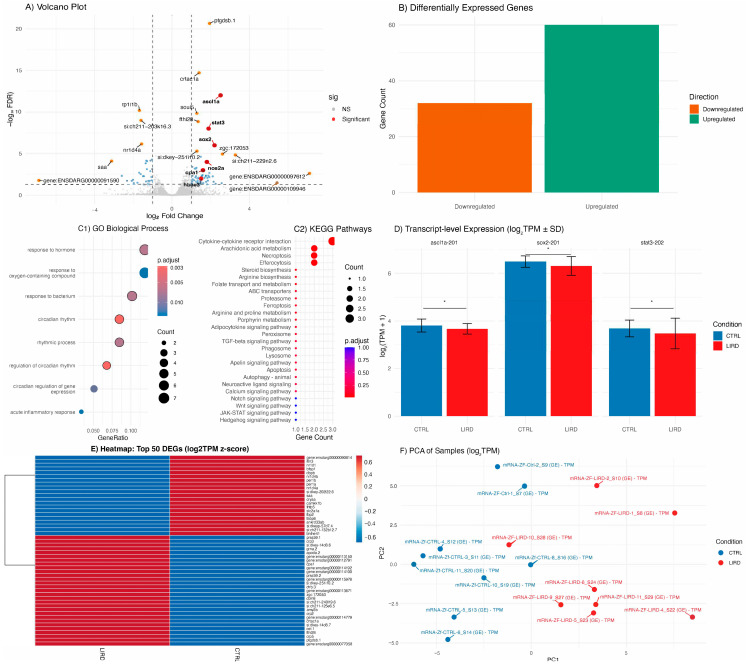
Transcriptomic responses to LIRD in zebrafish larvae. (**A**) Volcano plot showing the distribution of differentially expressed genes (DEGs) between control (CTRL) and LIRD retinae at 48 HPL. Genes are colored according to statistical significance and fold change. Selected regeneration-associated (*ascl1a*, *stat3*, *sox2*) and stress-related (*nos2a*, *cpa1*, *hbae3*) genes are highlighted. (**B**) Bar plot summarizing the number of significantly upregulated and downregulated DEGs identified between CTRL and LIRD conditions. (**C1**,**C2**) Bubble plots showing functional enrichment analyses of significantly regulated genes, including (**C1**) Gene Ontology (GO) Biological Process terms and (**C2**) KEGG signaling pathways. Dot size represents the number of genes per category, and color indicates adjusted *p*-values. (**D**) Transcript expression levels (log_2_(TPM + 1)) of selected isoforms (*ascl1a*-201, *sox2*-201, *stat3*-202), illustrating isoform-specific regulation following LIRD, * *FDR p*-value ≤ 0.05. (**E**) Heatmap showing expression patterns of the top 50 DEGs ranked by absolute fold change (z-scored log_2_ TPM). Red and blue indicate relative upregulation and downregulation, respectively, highlighting a distinct transcriptional signature in LIRD samples. (**F**) Principal component analysis (PCA) based on global gene expression (log_2_ TPM), showing clear separation between CTRL and LIRD groups. Each data point represents an independent biological replicate (N = 9 CTRL and 8 LIRD libraries, each library derived from a pool of 50 larval retinae).

To assess injury-associated transcriptional changes, bulk RNA-sequencing was performed on control and LIRD retinae collected 48 HPL. This analysis revealed a robust and reproducible differential transcriptomic response to phototoxic injury (Figure 3). Consistent with previously described molecular features of retinal injury and MG reprogramming [19,23,24,49,50,51] differential expression analysis identified upregulation of canonical regeneration-associated genes, including *ascl1a*, *stat3*, and *sox2* (Figure 3A,B,E).

Functional enrichment analyses based on Gene Ontology (GO) and KEGG pathway annotations highlighted significant regulation of signaling pathways previously implicated in MG activation and progenitor proliferation, including Notch, Wnt, JAK/STAT, and Hedgehog signaling (Figure 3C1,C2) [25,37,52]. In addition to gene-level changes, transcript-level quantification revealed isoform-specific regulation of selected regeneration-associated transcripts, including *ascl1a-201*, *stat3-202*, and *sox2-201* (Figure 3D), suggesting that phototoxic injury is accompanied by nuanced transcriptional regulation.

**Figure 4:**
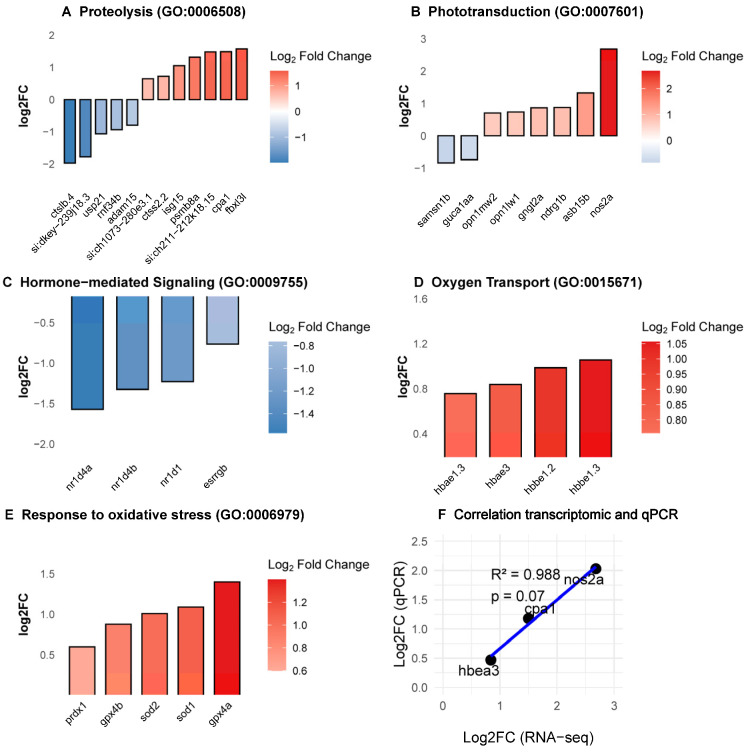
Underexplored biological processes engaged during the early larval LIRD response. (**A**–**E**) Functional enrichment analyses of differentially expressed genes (DEGs) highlight biological processes that are less commonly described in adult zebrafish retinal injury paradigms. (**A**) Proteolysis-related biological processes. (**B**) Phototransduction-associated processes. (**C**) Hormone-mediated signaling pathways, highlighted by coordinated downregulation of Rev-erb nuclear receptors (*nr1d1*, *nr1d4a*, *nr1d4b*). (**D**) Oxygen transport-associated genes, including hemoglobin subunits (*hbbe1.3*, *hbae3*, *hbbe1.2*, *hbae1.3*). (**E**) Response to oxidative stress. (**F**) RT–qPCR validation of selected DEGs (*cpa1*, *hbae3*, *nos2a*), confirming the directionality of expression changes observed by RNA sequencing. Data are presented as mean ± SEM. Each data point represents an independent biological replicate (N = 9 CTRL and 8 LIRD libraries, each library derived from a pool of 50 larval retinae).

In addition to canonical regeneration-associated pathways, functional enrichment analyses identified several biological processes that are less frequently discussed in the context of zebrafish retinal injury and regeneration. These include proteolysis, phototransduction remodeling, hormone-mediated signaling, oxygen transport, and response to oxidative stress (Figure 4A–E). RT-qPCR validation of selected differentially expressed genes (*cpa1*, *hbae3*, *nos2a*) confirmed the directionality of RNA-seq-derived expression trends (Figure 4F).

Proteolysis emerged as one of the most prominently enriched biological categories. Genes associated with proteolytic systems and post-translational regulation showed both up- and downregulation, suggesting a coordinated reorganization of proteostatic pathways following phototoxic injury (Figure 4A). Phototransduction remodeling-related processes were also significantly enriched (Figure 4B), which could indicate adaptive or compensatory transcriptional responses following photoreceptor stress. Hormone-mediated signaling pathways were similarly enriched, driven primarily by coordinated downregulation of Rev-erb nuclear receptors (*nr1d4a*, *nr1d4b*, *nr1d1*; Figure 4C). Unexpectedly, genes encoding hemoglobin subunits (*hbbe1.3*, *hbae3*, *hbbe1.2*, and *hbae1.3*) were among the most strongly upregulated transcripts within the oxygen transport category (Figure 4D). While hemoglobins are classically associated with erythroid cells, increasing evidence supports non-canonical roles for hemoglobin expression in redox buffering and nitric oxide signaling in non-hematopoietic tissues [53,54,55] Consistent with this observation, the response to oxidative stress was significantly enriched, with increased expression of several antioxidant and redox-regulatory genes, including *prdx1*, *sod1*, *sod2*, *gprx4a*, *gprx4b* (Figure 4E). Taken together, acute LIRD engages a broad transcriptional stress-response program that extends beyond classical regeneration-associated pathways.

### 3.3. Oxidative Stress Influences Regeneration-Associated Responses

The robust transcriptional regulation of oxidative stress- and antioxidant-associated gene networks observed following larval LIRD, including detoxification enzymes and hemoglobin-related genes, prompted us to functionally probe the contribution of oxidative stress to early regeneration-associated responses following phototoxic injury.

**Figure 5:**
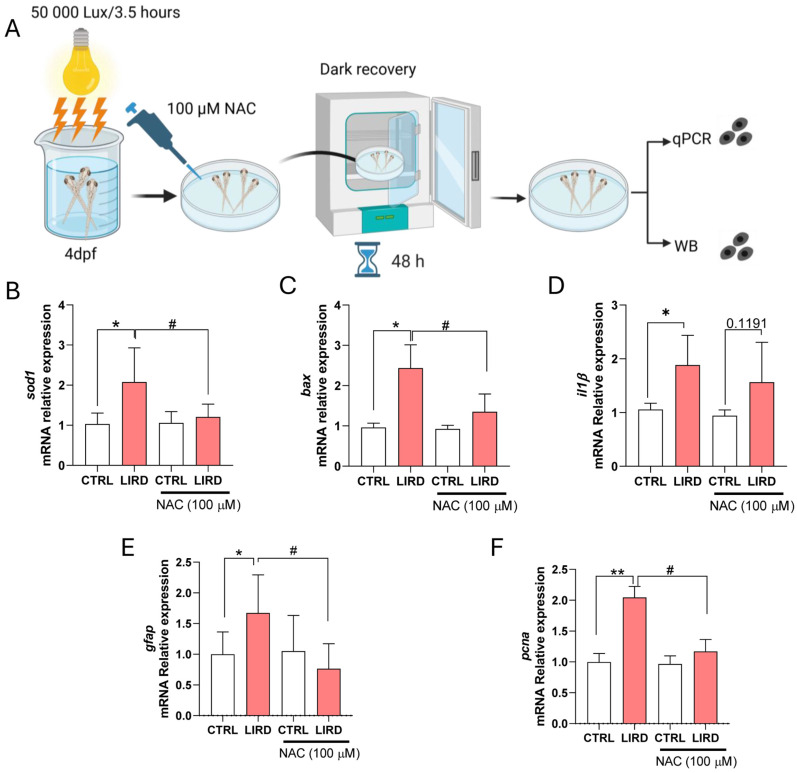
Antioxidant treatment modulates oxidative stress, apoptosis, and early regeneration-associated responses following LIRD. (**A**) Schematic overview of the LIRD paradigm and NAC treatment. Immediately following LIRD, larvae were transferred to E3 medium containing 100 μM *N*-acetylcysteine (NAC) and allowed to recover for 48 h under standard conditions. Pools of 50 larval retinae were collected for each biological replicate for RT–qPCR or WB analyses. Created in BioRender. Poggi, L. (2026) https://BioRender.com/fpmmeyk (**B**–**F**) RT–qPCR analysis of oxidative stress, apoptosis, inflammation, and regeneration-associated markers. Expression levels of *sod1* (**B**), *bax* (**C**), *il1β* (**D**), *gfap* (**E**), and *pcna* (**F**) were normalized to the housekeeping gene *ube2a* and expressed as fold change relative to control. N = 4 biological replicates per condition. Data are presented as mean ± SD. Statistical significance was assessed using two-way ANOVA with Sidak’s multiple-comparisons test. * *p* ≤ 0.05, ** *p* ≤ 0.01, # *p* ≤ 0.05, as indicated.

To this end, larvae were allowed to recover for 48 h after LIRD in the presence of the antioxidant *N*-acetylcysteine (NAC) (Figure 5A). RT–qPCR analysis confirmed that expression of the oxidative stress-responsive gene *sod1* was strongly induced following LIRD and that this induction was significantly attenuated by NAC treatment (Figure 5B), consistent with effective modulation of oxidative stress under these conditions. Similarly, expression of the pro-apoptotic gene *bax* was elevated after LIRD and significantly reduced in NAC-treated larvae (Figure 5C), indicating suppression of apoptosis-associated signaling. In contrast, expression of the inflammatory marker *il1β* remained elevated following LIRD and was not reduced by NAC treatment (Figure 5D), suggesting that inflammatory activation is largely maintained independently of antioxidant-mediated modulation of oxidative stress in this paradigm.

We next examined markers associated with early regeneration-related responses. LIRD induced a significant increase in *gfap* expression, which was attenuated by NAC treatment (Figure 5E). In parallel, expression of the proliferation-associated marker *pcna* was significantly reduced in NAC-treated larvae compared with LIRD alone (Figure 5F). Together, these data suggest that oxidative stress contributes to the induction or maintenance of early glial activation and proliferation-associated transcriptional responses following phototoxic injury.

**Figure 6:**
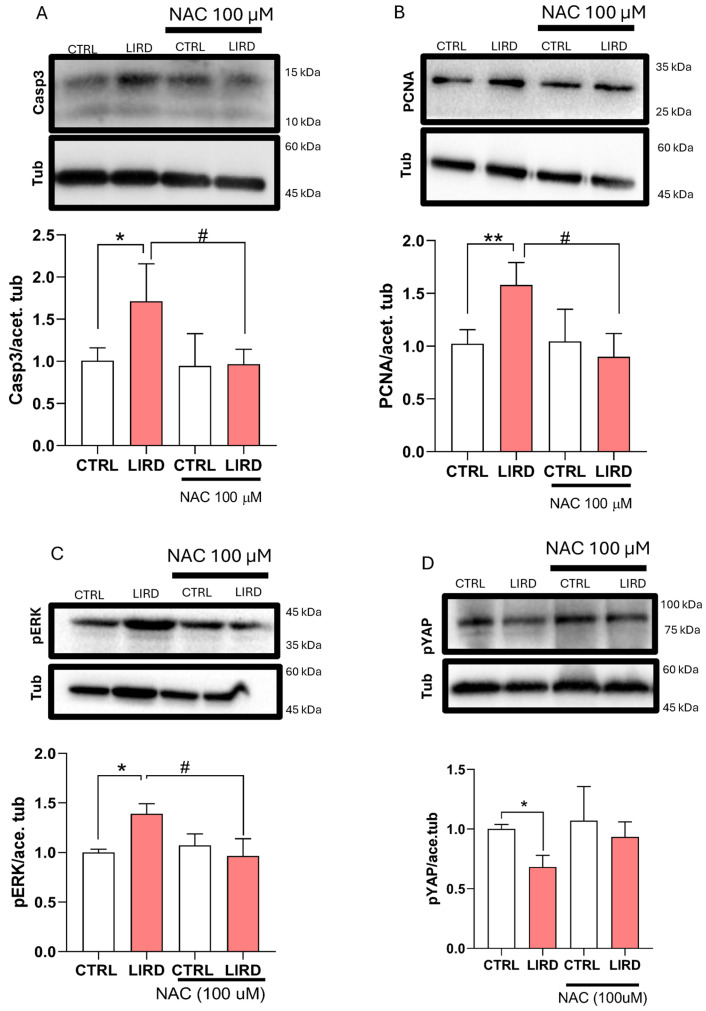
Antioxidant treatment modulates apoptosis- and growth-associated signaling following LIRD. (**A**–**D**) WB analyses assessing oxidative stress-associated responses following LIRD in the presence or absence of NAC. (**A**) Representative WB (top) showing cleaved caspase-3 (Casp3), with acetylated tubulin (Tub) used as a loading control. Densitometric quantification (bottom) is normalized to control levels (N = 4 biological replicates per condition, each derived from a pool of 50 larval retinae). (**B**) Representative WB (top) showing proliferating cell nuclear antigen (PCNA), with acetylated tubulin used as a loading control. Densitometric quantification (bottom) normalized to control levels (N = 4 biological replicates per condition). (**C**) Representative WB (top) showing phosphorylated ERK (pERK), with acetylated tubulin used as a loading control. Densitometric quantification (bottom) normalized to control levels (N = 4 biological replicates per condition). (**D**) Representative WB (top) showing phosphorylated YAP (pYAP), with acetylated tubulin used as a loading control. Densitometric quantification (bottom) normalized to control levels (N = 4 biological replicates per condition). Data are presented as mean ± SD. Statistical significance was assessed using two-way ANOVA with Sidak’s multiple-comparisons test. * *p* ≤ 0.05, ** *p* ≤ 0.01, # *p* ≤ 0.05, as indicated.

To validate the transcriptional effects of antioxidant treatment at the protein level, we performed WB analyses on larval retinae collected 48 h after LIRD in the presence or absence of *N*-acetylcysteine (NAC) (Figure 6). Consistent with the induction of apoptosis-associated pathways following phototoxic injury, LIRD resulted in a marked increase in cleaved caspase-3 protein levels, which was significantly attenuated by NAC treatment (Figure 6A). In parallel, the LIRD-induced elevation of proliferating cell nuclear antigen (PCNA) was reduced in NAC-treated larvae, indicating that proliferation-associated responses were suppressed under antioxidant conditions (Figure 6B).

Key growth- and regeneration-associated signaling components, including the Hippo and Mitogen-Activated Protein Kinase (MAPK) pathways, were enriched in our transcriptomic analysis and have been previously implicated in retinal injury and regeneration [10,56,57] We therefore examined whether mitigation of oxidative stress influences these pathways. LIRD exposure was associated with increased levels of phosphorylated ERK (pERK) and reduced levels of phosphorylated YAP (pYAP), consistent with modulation of MAPK- and Hippo-associated signaling. Both responses were significantly attenuated by NAC treatment (Figure 6C,D). Together, these data indicate that NAC treatment modulates apoptosis- and growth-associated signaling responses induced by LIRD.

### 3.4. Subtoxic H_2_O_2_ Exposure Promotes Proliferation in the Absence of Overt Apoptosis

**Figure 7:**
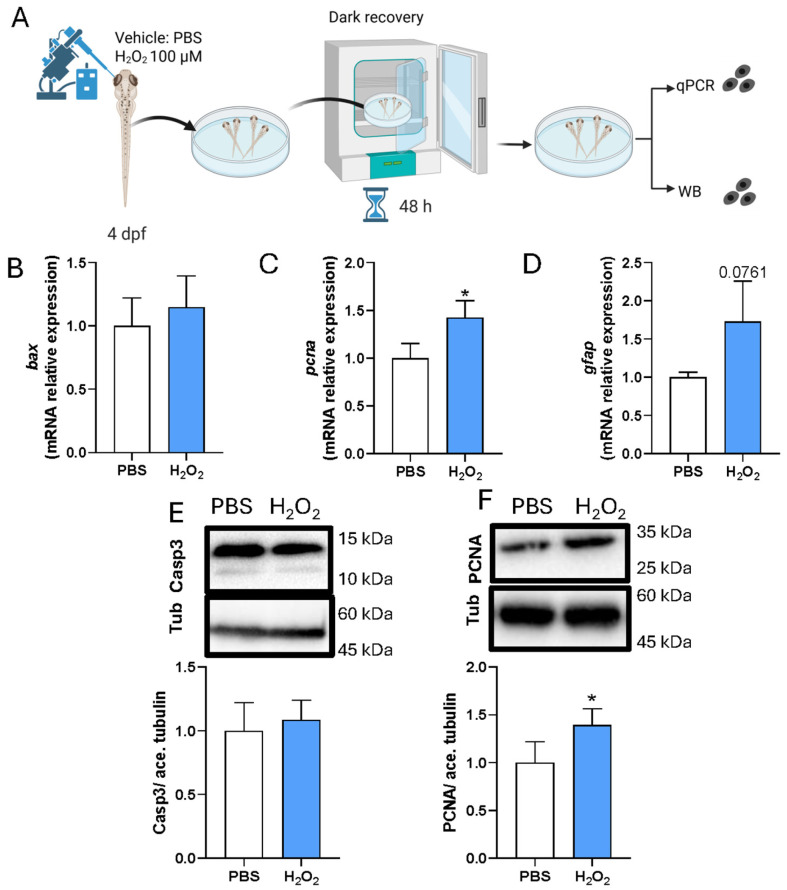
Subtoxic H_2_O_2_ exposure promotes proliferation without overt apoptosis in zebrafish larvae. (**A**) Schematic of intra-retinal injection of vehicle (PBS) or hydrogen peroxide (H_2_O_2_; 100 μM) in 4 dpf zebrafish larvae, followed by 48 h recovery under standard conditions. Pools of 50 larval retinae were collected for RT–qPCR or WB analysis, Created in BioRender. Poggi, L. (2026) https://BioRender.com/v1i9rhx. (**B**–**D**) RT–qPCR analysis of *bax* (**B**), *pcna* (**C**), and *gfap* (**D**), normalized to the housekeeping gene *ube2a* and expressed relative to PBS controls. (**E**) Top: Representative WB showing cleaved caspase-3 with acetylated tubulin as a loading control. Bottom: Densitometric quantification normalized to control. (**F**) Top: Representative WB showing PCNA with acetylated tubulin as a loading control. Bottom: Densitometric quantification normalized to control. N = 4 biological replicates per condition, each consisting of pooled larval retinae. Data are shown as mean ± SD. Statistical significance was assessed by unpaired *t*-test; * *p* ≤ 0.05.

Given the reduction in proliferation-associated responses observed upon antioxidant treatment following LIRD, we next asked whether a moderate increase in ROS levels is sufficient to influence proliferation-associated markers in the absence of phototoxic injury. To address this, subtoxic intra-retinal injections of vehicle (PBS) or hydrogen peroxide (H_2_O_2_) were performed in 4 dpf larvae, which were then allowed to recover under standard conditions for 48 h (Figure 7A).

RT–qPCR analysis showed that expression of the pro-apoptotic gene *bax* was not significantly altered following H_2_O_2_ injection compared with PBS controls, indicating the absence of a detectable apoptotic transcriptional response under these conditions (Figure 7B). In contrast, *pcna* expression was significantly increased, while *gfap* showed a modest but non-significant upward trend (Figure 7C,D), consistent with the engagement of proliferation-associated programs without robust glial activation. Protein-level analyses corroborated these findings. WB analysis confirmed that H_2_O_2_ exposure did not induce caspase-3 cleavage, supporting the absence of overt apoptosis, whereas PCNA protein levels were significantly increased relative to controls (Figure 7E,F).

Together, these results indicate that subtoxic elevation of ROS is sufficient to enhance proliferation-associated markers in the larval zebrafish retina, in the absence of phototoxic injury or detectable apoptosis.

## 4. Discussion

Several paradigms have been used to study the remarkable regenerative capacity of the zebrafish retina, including physical lesions, chemical ablation, and light-induced retinal damage (LIRD) [14,24,26,29,31,58,59,60] While LIRD is well-characterized in adults, few studies have examined larval stages [25,37].

In this study, we established and validated a pigmented larval LIRD model that recapitulates key features of early degeneration and regeneration. Within 48 h post-lesion, apoptotic pathways (*bax*, cleaved caspase-3) were activated alongside markers of MG reactivity and proliferation (GFAP, PCNA).

Histological assessment of the photoreceptor cell layer (PCL) revealed an acute stress response. Although DAPI/Zpr1+) photoreceptor cells remained structurally present, the apical-basal collapse of the Zpr1+ domain, and the induction of apoptotic markers suggest these cells initiate a pathological stress program prior to overt loss, consistent with adult LIRD paradigms [15,61] Likewise, we observed molecular up-regulation of GFAP and the emergence of mCherry+/PCNA+ cells (validated as predominantly GS+) in the central retina. Notably, activated MG do not immediately downregulate differentiation markers upon cell-cycle re-entry, but instead transition through a GS+/PCNA+ state before undergoing full dedifferentiation [6,35]. Thus, the presence of PCNA+ MG in our model serves as a definitive marker of an injury-induced regenerative program.

Transcriptomic profiling revealed induction of canonical regeneration-associated genes, including *ascl1a*, *stat3*, and *sox2*, reflecting early MG dedifferentiation and progenitor activation [23,27,62]. Beyond canonical pathways, our analysis highlighted an array of underexplored processes as well as isoform-specific regulation, underscoring the complex landscape of the phototoxic injury response.

Genes involved in proteolysis and extracellular matrix (ECM) remodeling, including *cpa1* and the immunoproteasome subunit *psmb8a*, were significantly upregulated. These changes suggest dynamic proteostasis and ECM restructuring during the early injury response. While ECM remodeling remains poorly characterized in the regenerating retina, evidence from zebrafish spinal cord injury demonstrates that ECM dynamics facilitate neural restoration through interactions with Wnt signaling [63]. In line with this, the enrichment of Wnt-related pathways in our KEGG analysis raises the possibility that ECM remodeling and Wnt activation are coordinately engaged during early retinal repair. Such remodeling may not only support MG nuclear migration and progenitor expansion, but also prevent fibrotic scarring, thereby preserving regenerative competence [64].

Our dataset also revealed a complex sensory-transduction signature characterized by bidirectional regulation: suppression of phototransduction-reset genes alongside upregulation of multiple cone opsins. Rather than reflecting simple photoreceptor shutdown, this transcriptional reorganization may indicate early circuit remodeling or compensatory adaptation following phototoxic stress. Zebrafish photoreceptors become post-mitotic and fully functional by 72 hpf, even if they continue to refine their structure and sensitivity throughout the larval period [65,66]. It is therefore tempting to speculate that the selective regulation of distinct opsin genes reflects MG re-engaging in lineage-specific photoreceptor developmental programs [67,68]. Distinguishing this regenerative potential from a secondary adaptive stress response will, however, require functional validation.

Furthermore, the coordinated downregulation of Rev-erb nuclear receptors (*nr1d1*, *nr1d4a*, *nr1d4b*) points to the contribution of circadian and metabolic regulatory networks. As key integrators of rhythmic timing and homeostasis, Rev-erbs are essential for retinal differentiation and survival [69,70,71]. Their suppression following LIRD may signal metabolic reprogramming necessary to unlock MG plasticity, perhaps by altering energy utilization to support the transition from a quiescent to a proliferative state. Alternatively, given their known neuroprotective functions, loss of Rev-erbs might instead exacerbate the retina’s vulnerability to phototoxic stress [72].

A central finding of this study is the contribution of oxidative stress to early retinal responses. LIRD induced upregulation of antioxidant enzymes and non-canonical hemoglobin subunits (*hbbe1.3*, *hbae3*, *hbbe1.2*, *hbae1.3*). Although classically associated with erythroid lineages, hemoglobins are increasingly recognized for their non-canonical roles in redox buffering and nitric oxide signaling in non-hematopoietic tissues, including the brain [53,54,55,73]. Whether this signature represents de novo endogenous expression by retinal cells as a neuroprotective response, or the recruitment of transcriptionally active erythroid cells remains to be determined. Nevertheless, it reinforces the prominence of oxidative stress in the early LIRD landscape, highlighting a compelling new avenue for research into the larval LIRD stress response.

We sought to functionally dissect the relationship by pharmacologically modulating ROS in the LIRD larval model. Scavenging ROS with NAC attenuated apoptotic signaling (reduced cleaved caspase-3 and *bax*) and suppressed MG proliferation (PCNA and *gfap*), without significantly affecting the inflammatory marker *il1β*. This apparent dissociation suggests that, in this early injury phase or within a specific temporal window, ROS may function as a permissive signal for MG activation rather than as a primary driver of the broader inflammatory cascade. This is further supported by our observation that low-dose H_2_O_2_ exposure induced MG proliferative markers without eliciting apoptosis. Collectively, these findings suggest a “functional redox window” [74], wherein ROS serves as mitogenic signals for MG below the threshold required to trigger programmed cell death or amplify the broader inflammatory cascade.

While NAC shows clinical promise in Retinitis Pigmentosa [75], its in vivo activity is complex and dose dependent [76,77]. As we utilized a single concentration, future studies are required to define the specific temporal dynamics, and potential ROS-independent mechanisms involved. Such investigations will be essential to clarify the role of antioxidant-based modulation in the regenerating retina.

Notably, the conditions tested of H_2_O_2_ exposure resulted in a modest reduction in phosphorylated YAP (*p* = 0.08), while phosphorylated ERK levels remained unchanged (see Supplementary Figure S1). These observations suggest that proliferation induced by subtoxic ROS may occur independently of robust ERK/MAPK or Hippo/YAP pathway activation, or alternatively, may reflect the transient nature of a single H_2_O_2_ exposure. During LIRD, ROS are likely produced continuously, potentially engaging multiple signaling pathways over time. Therefore, future experiments utilizing repeated or sustained ROS elevation might be required to fully clarify their contribution to the proliferative response. Collectively, these data position ROS as a modulatory axis within the early retinal injury milieu, consistent with observations in other regenerative contexts, such as zebrafish fin regeneration [78,79]. However, these findings do not yet establish ROS as singular drivers of regeneration, nor do they resolve whether they act upstream, downstream, or in parallel with inflammatory pathways.

Several technical limitations also warrant consideration. Bulk RNA-seq cannot resolve cell-type-specific or spatially restricted transcriptional responses, and isoform-level dynamics remain correlative rather than mechanistic. While our transcriptomic analysis reveals the robust activation of canonical regenerative factors, including *ascl1a*, *stat3*, and *sox2*, further functional validation via genetic loss-of-function studies is required to establish their causal roles in MG dedifferentiation in this model. Similarly, the observed changes in ECM remodeling, proteolysis, and oxygen transport represent a complex stress response that may be either permissive for regeneration or secondary to phototoxic injury. Additionally, while the larval LIRD paradigm serves as a high-throughput platform for pharmacological screening, it utilizes an acute, high-intensity insult that differs from the chronic, progressive nature of human retinal degenerations.

In summary, this study establishes the larval LIRD model as a tractable platform for interrogating early cellular responses to phototoxic and oxidative injury. We provide a comprehensive transcriptomic resource and demonstrate a functional contribution of ROS to MG activation during the early stress response. These findings lay the groundwork for future investigations aimed at defining the ‘functional redox window’ and elucidating the interplay between ROS-mediated signaling and canonical regenerative pathways.

## Data Availability

All relevant data supporting the findings of this study, including quantitative analyses and representative images, are provided in the main text and Supplementary Information, or will be made available upon request. All raw and processed transcriptomic data supporting the conclusions of this work have been submitted to the NCBI Gene Expression Omnibus (GEO) and are available under the GEO accession number GSE313277.

## Supplementary Materials

The following supporting information can be downloaded at: https://www.mdpi.com/article/10.3390/antiox15030348/s1, Table S1: Antibodies; Table S2: Primers, Supplementary Figure S1: Larval Light-Induced Retinal Damage, Created in BioRender. Poggi, L. (2026) https://BioRender.com/fpmmeyk, Supplementary Figure S2: Fli labels most of the Müller glia (MG) in the inner retinal layer, Supplementary Figure S3: Active caspase 3 immunolabeling, Supplementary Figure S4: Effects of H_2_O_2_ on Hippo and ERK pathway activity.

## Abbreviations

The following abbreviations are used in this manuscript:

BP	Biological process
CC	Cellular component
DEG	Differentially expressed genes
dpf	Days post-fertilization
GCL	Ganglion cell layer
GFAP	Glial Fibrillary Acidic Protein
HPL	Hours-post-light-induced retinal damage
INL	Inner nuclear layer
LIRD	light-induced retinal damage
MF	Molecular function
MG	Müller glia
NAC	*N*-acetylcysteine
NO	Nitric oxide
PCA	Principal component analysis
PCL	Photoreceptor cell layer
PCNA	proliferating cell nuclear antigen
ROS	Reactive oxygen species
TPM	Transcripts per million
WB	Western Blot
