# Supplementary material for: Oxidative Stress Signaling and Regenerative Responses in a Larval Zebrafish Model of Retinal Light Damage
Source: Antioxidants (Basel). 2026 Mar 10;15(3):348. doi: 10.3390/antiox15030348 (PMC13023554; doi:10.3390/antiox15030348)
Supplement: Supplementary file 1 [file antioxidants-15-00348-s001.zip › Supplementary information Babiloni-Chust. et al.pdf]

Supplementary Table S1: Antibodies

Antibody	Aplication	Dilution	Manufacturer	Reference
**PCNA**	**WB/IHC**	1:750/1:500	Calbiochem	NA03-200UG
**GFAP**	**WB**	1:2000	Dako	ZO334
**Active Casp3**	**WB/IHC**	1:750/1:500	Abcam	AB13847
**pERK**	**WB**	1:750	Santa Cruz	SC-16982
**pYAP**	**WB**	1:500	Cell Signaling Technology	#4911
**Acetylated Tubulin**	**WB**	1:1000	Thermo Fisher Scientific	32-2700
**Actin**	**WB**	1:1000	Santa Cruz	sc-1616
**ZPR1**	**IHC**	1:500	ZIRC	
**GS**	**IHC**	1:500	Millipore	MAB302
**goat anti-mouse IgG-HRP**	**WB**	1:5000	Santa Cruz Biotechnology	sc-2005
**goat anti-rabbit IgG-HRP**	**WB**	1:5000	Santa Cruz Biotechnology	sc-2004
**mouse anti-goat IgG-HRP**	**WB**	1:5000	Santa Cruz Biotechnolog	sc-2354
**Goat Alexa Fluor 488 anti-Mouse**	**IHC**	1:1000	Invitrogen	A11001

WB: Western Blot, IHC: immunohistochemistry

Supplementary Table S2: Primers

Gene	Forward primer (5`-3`)	Reverse primer (5`-3`)
cpa1	CCATGCTAGCGGAATCTGGT	CCAGGATTGGGCTTTCTGGT
hbae3	CGCAAAGGACAAAGCGAACG	CGTGGTTCCGTGCTTCTTCA
nos2a	TGGAGAAATGCGCCAAGATG	CCGTTGGTGGCATACTTCAG
sod1	GAACCACAGGGTGAGCTGTT	GTTAGCACACGCTGCAATTCC
bax	CTGGCAGACAGTCGGAGTTT	GAAGCGGTTTCACCTCTCAA
il1β	TTTGTGGGAGACAGACGGTG	TCAGGGCGATGATGACGTTC
gfap	ACCCGTGACGGAGAGATCAT	GCCAGTGTCTGAGCCTCATT
pcna	CAAGGAGGATGAAGCGGTAACA	CTGCGGACATGCTAAGTGTG
ube2a	TGACTGTTGACCCACCTTACAG	CAAATAAAAGCAAGTAACCCC

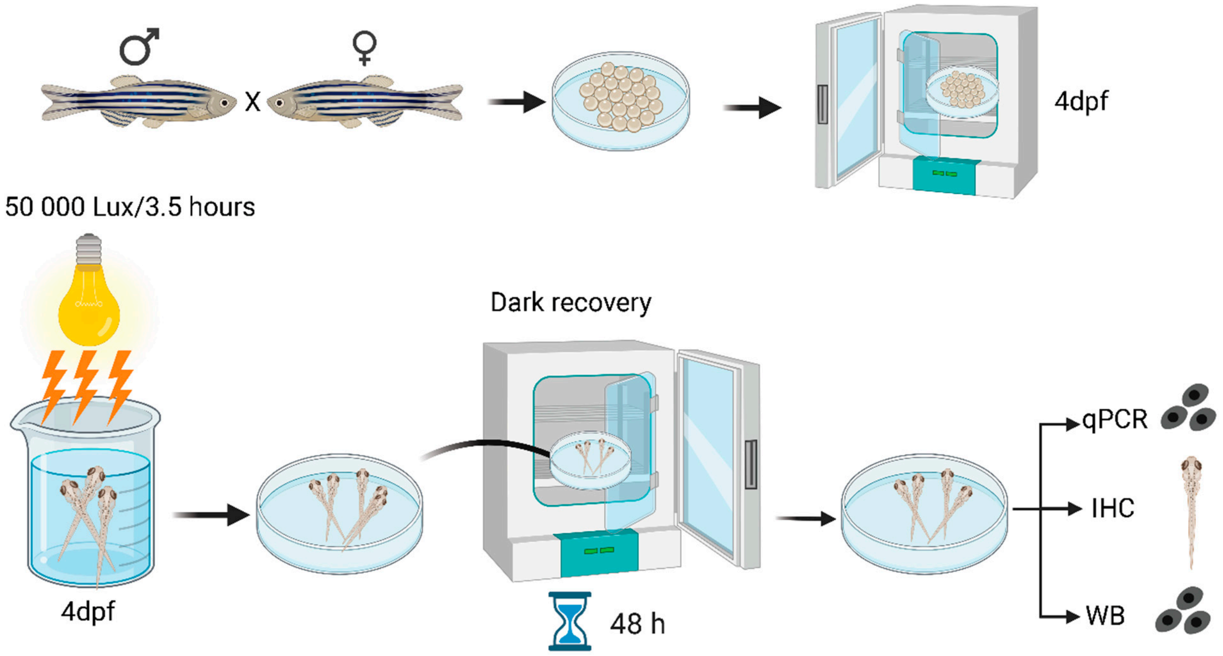

Supplementary Figure 1: Larval Light-Induced Retinal Damage. Natural crosses between adult zebrafish were performed, eggs collected and incubated at 28ºC. At 4 days post-fertilization (dpf) pigmented zebrafish larvae were exposed to high-intensity white light (50,000 lux) for 3.5 h, followed by a 48-h recovery period. The downstream analysis were Western Blot (WB), immunohistochemistry (IHC) and bulk-RNA sequencing. Created with BioRender.com

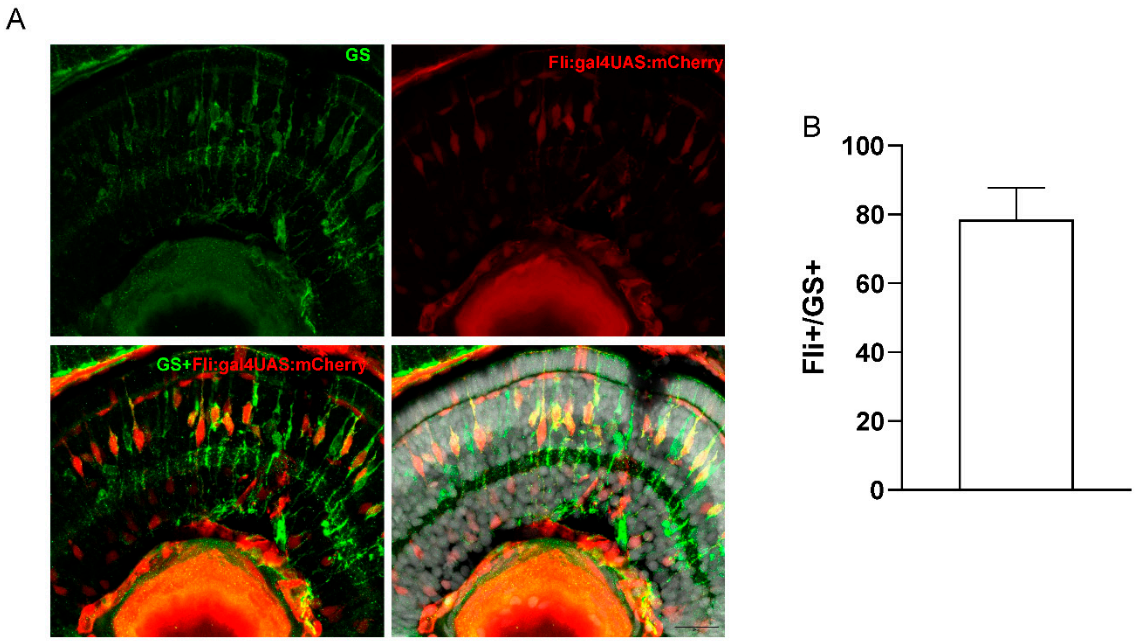

Supplementary Figure 2: Fli labels most of the Müller glia (MG) in the inner retinal layer. (A) Representative confocal images of 6 dpf retinas immunolabeled with the Müller glia marker GS (green). Upper right: GS staining. Upper left: Fli:gal4UAS:mCherry (red). Bottom right: merge of GS and Fli:gal4UAS:mCherry. Bottom left: merge of GS, Fli:gal4UAS:mCherry and DAPI (nuclei, grey). (B) Quantification of Fli^+/GS^+ cells, expressed as percentage relative to the total number of GS^+ cells (GS = 100%). Data are presented as mean ± S.D. (n = 7)

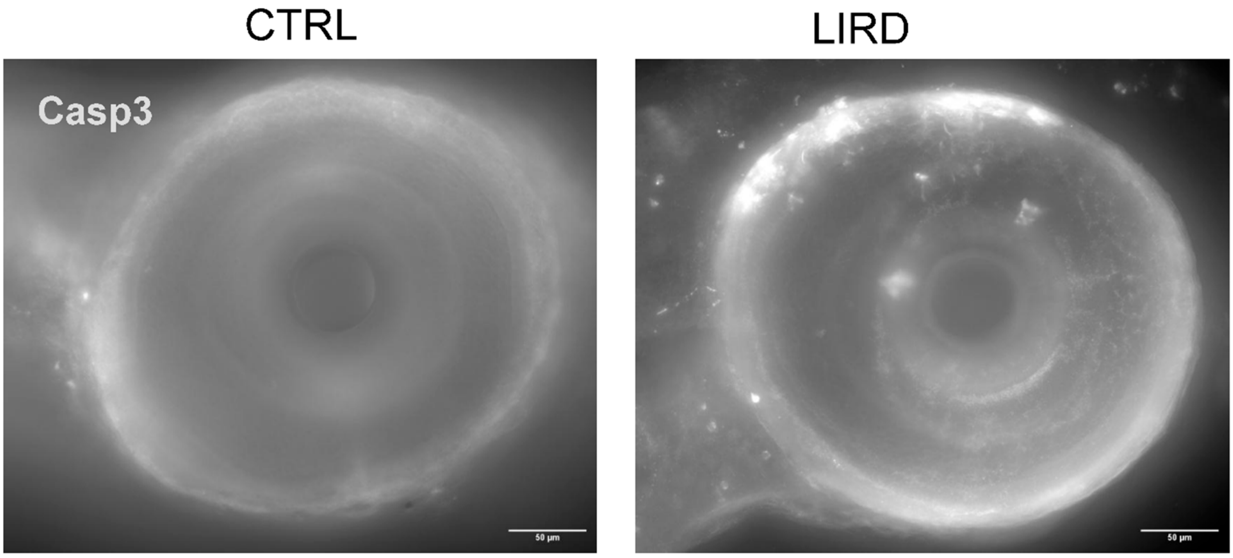

Supplementary Figure 3: Active caspase 3 immunolabeling. After 48 hours of the LIRD, embryos were fixed and immunolabeled against cleaved caspase 3 (casp3).

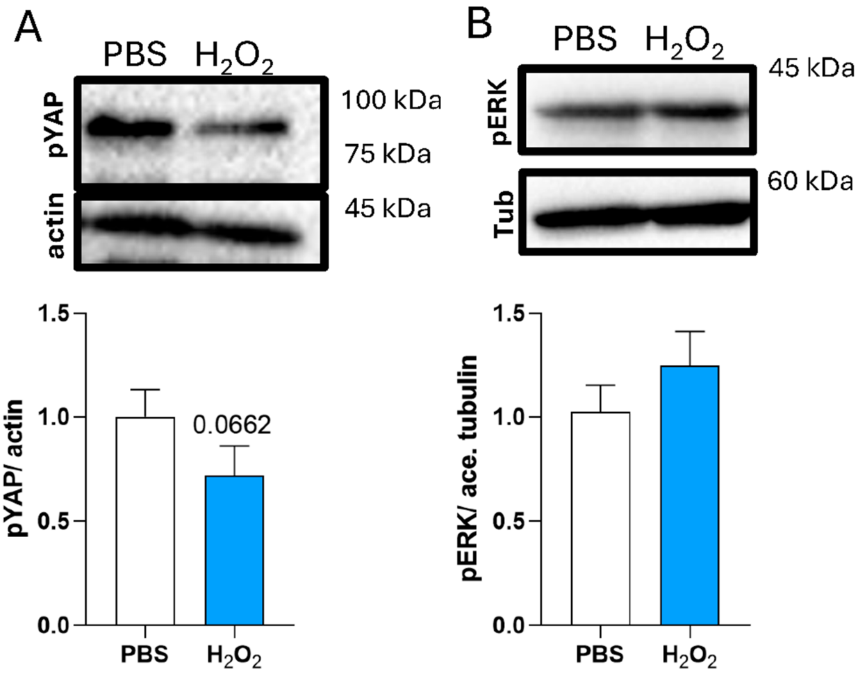

Supplementary Figure 4: Effects of H2O2 on Hippo and ERK pathway activity. (A) Western blot analysis of phosphorylated YAP (pYAP). (B) Western blot analysis of phosphorylated ERK (pERK). Densitometric quantification normalized to control averages; N = 4 biological replicates; mean ± s.d.; unpaired t-test.
