# Supplementary figures and images for: Oxidative Stress Signaling and Regenerative Responses in a Larval Zebrafish Model of Retinal Light Damage
Source: Antioxidants (Basel). 2026 Mar 10;15(3):348. doi: 10.3390/antiox15030348 (PMC13023554; doi:10.3390/antiox15030348)

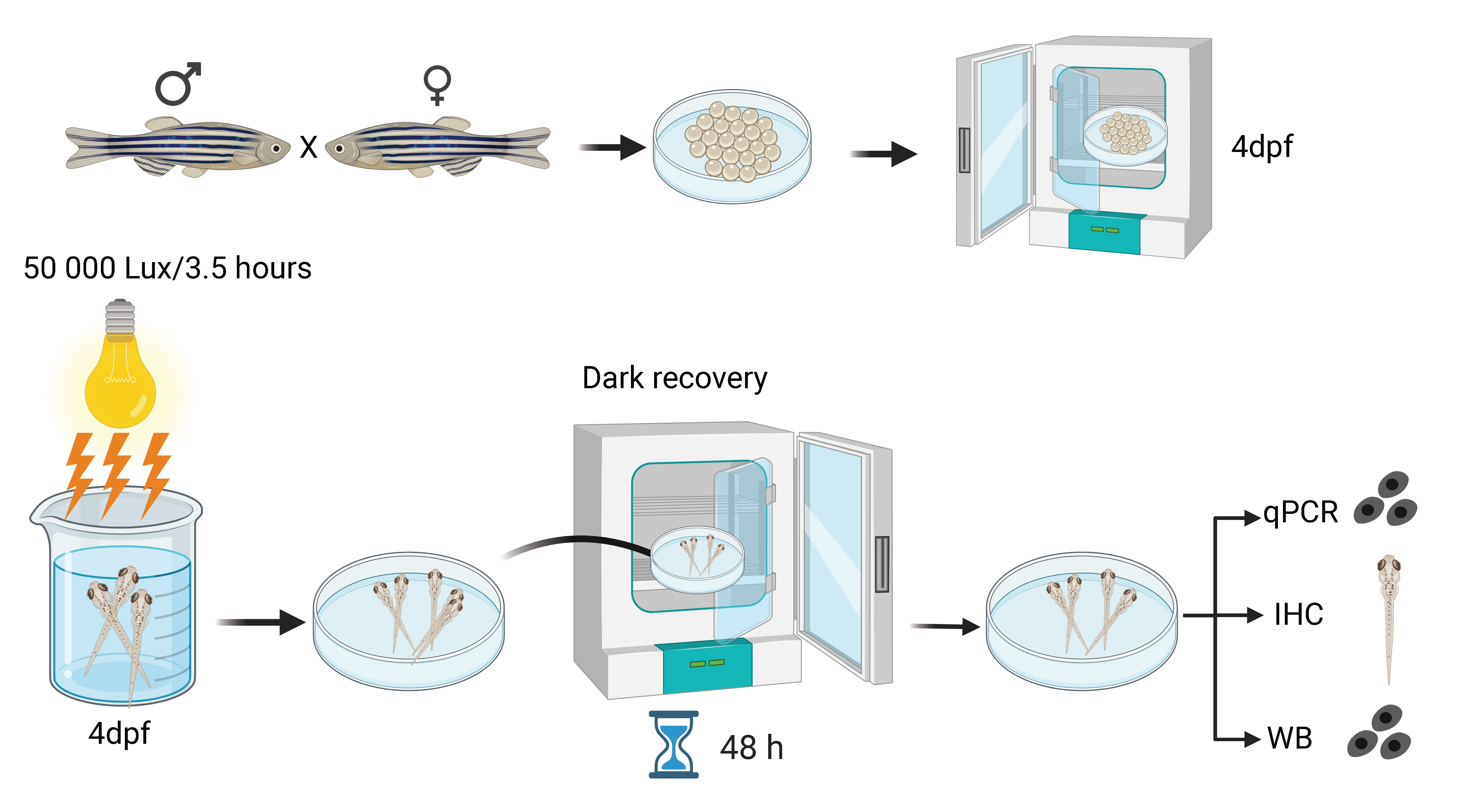

Supplement: Supplementary file 1 [file antioxidants-15-00348-s001.zip › SUPP FIGURE 1.tif]

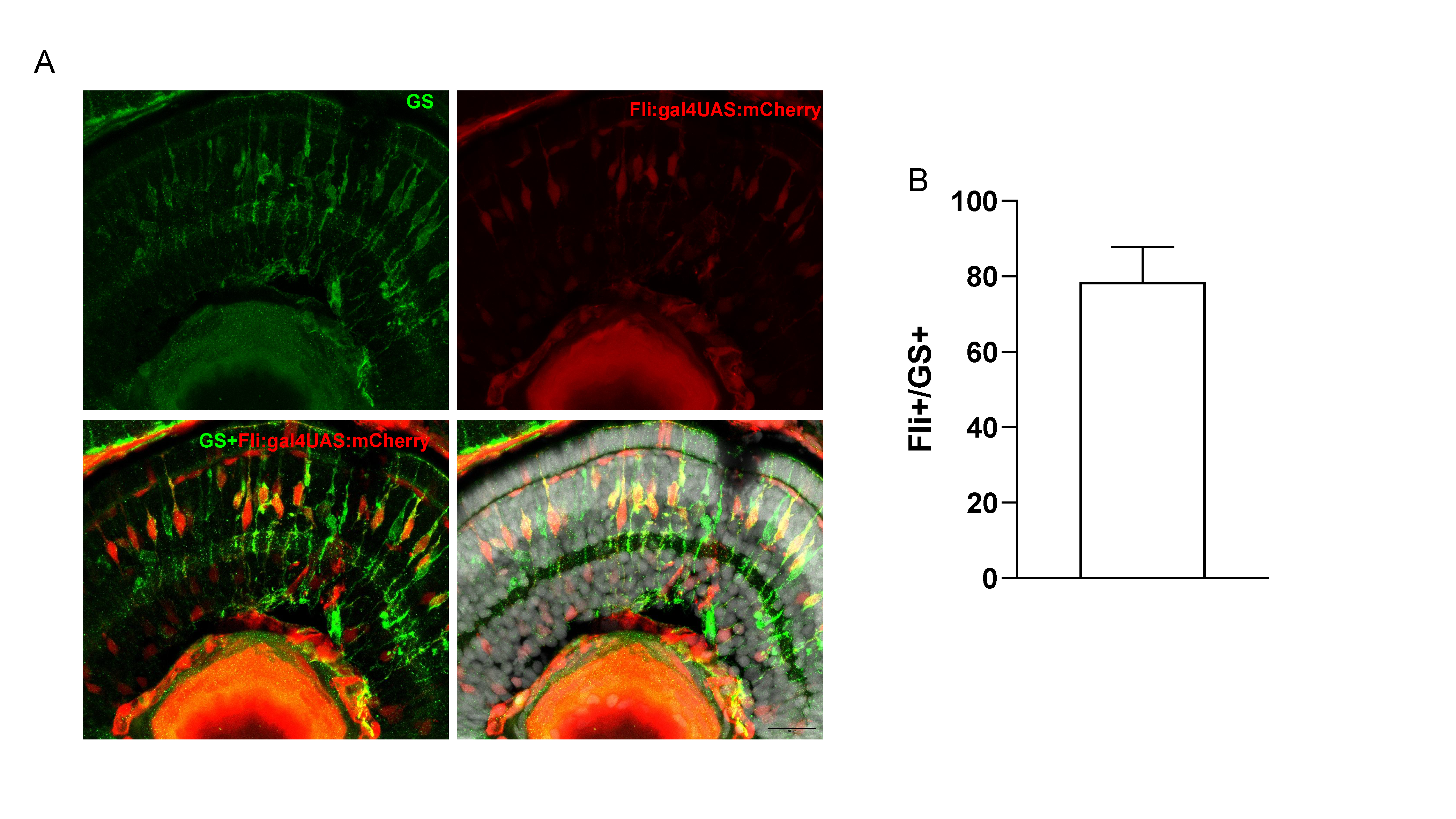

Supplement: Supplementary file 1 [file antioxidants-15-00348-s001.zip › SUPP FIGURE 2.tif]

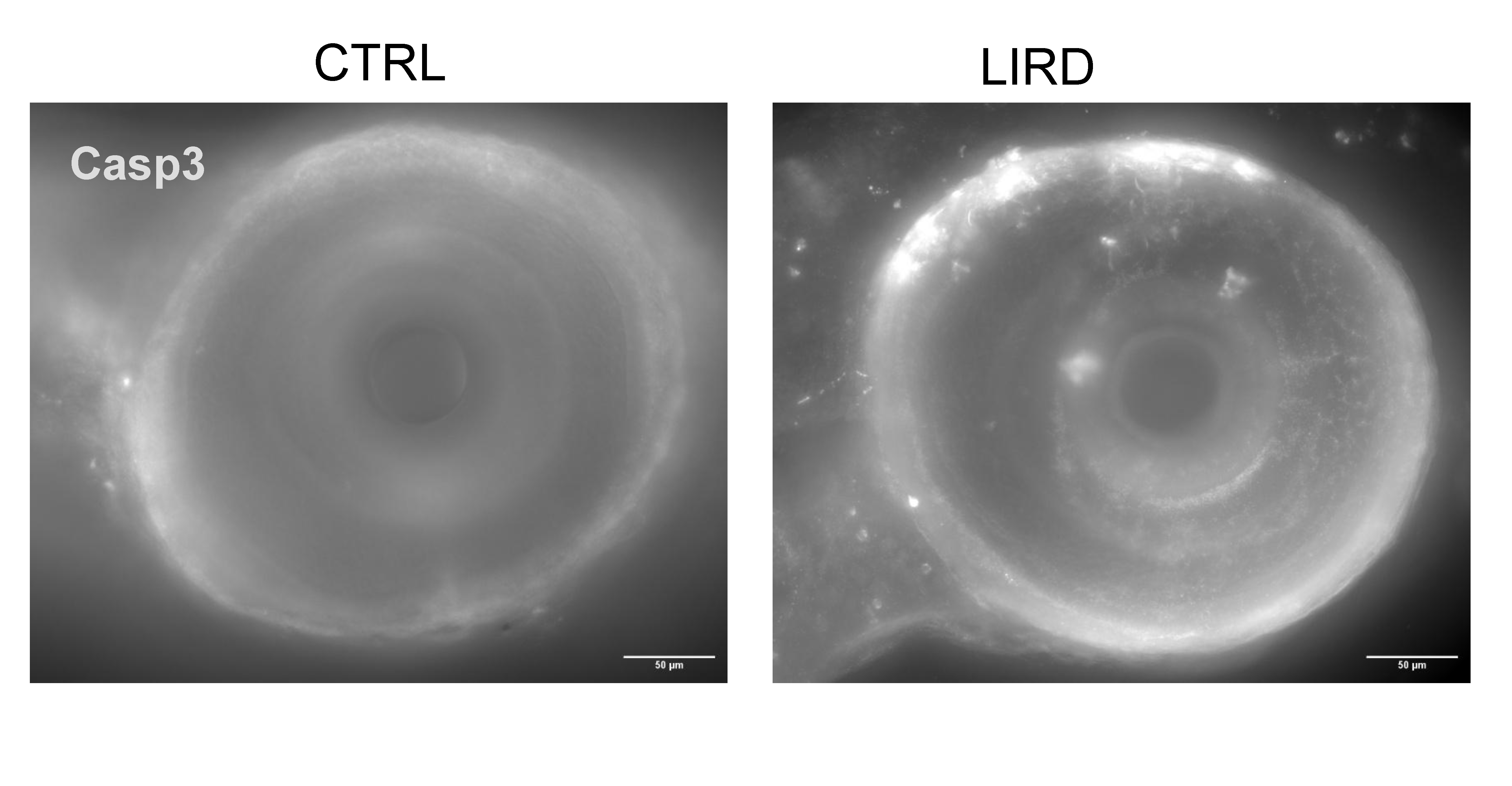

Supplement: Supplementary file 1 [file antioxidants-15-00348-s001.zip › SUPP FIGURE 3tif.tif]

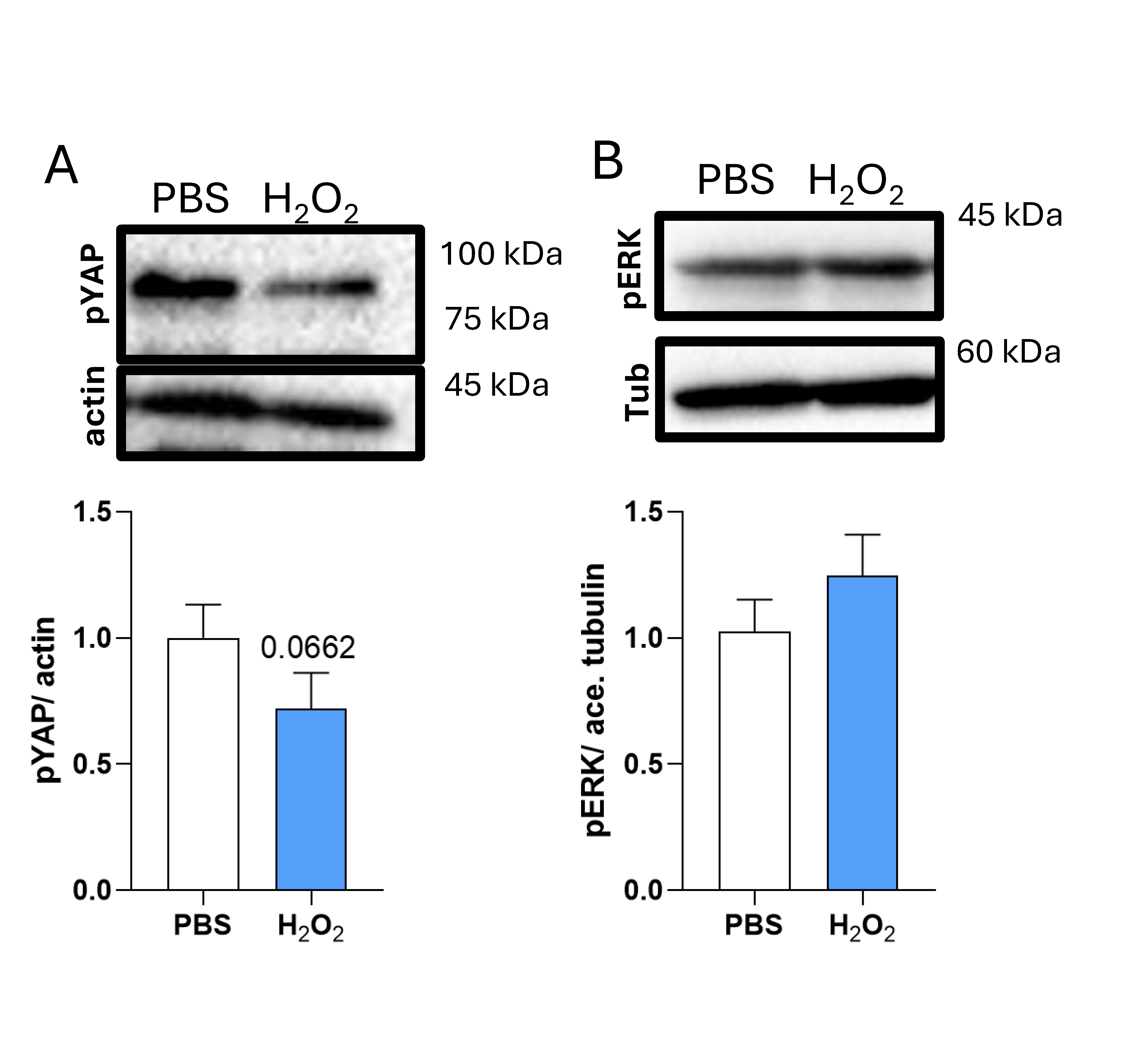

Supplement: Supplementary file 1 [file antioxidants-15-00348-s001.zip › SUPP FIGURE 4.tif]
